# Gallstone-Related Complication 11 Years After Laparoscopic Cholecystectomy: A Report of a Rare Case

**DOI:** 10.7759/cureus.116928

**Published:** 2026-09-25

**Authors:** Alaa Kansoun, Nicolas Turrin, Sami Hamed, Xavier Hanna

**Affiliations:** 1 Digestive and Endocrine Surgery, Hôpital Saint Joseph, Marseille, FRA

**Keywords:** dropped gallstone, gallbladder diseases and gallstones, gallstone spillage, laparoscopic cholecystectomy, residual gallstone disease

## Abstract

Gallbladder perforation during laparoscopic cholecystectomy is relatively common and may result in bile and gallstone spillage. Although most retained gallstones remain clinically silent, they can rarely serve as a nidus for delayed intra-abdominal or abdominal wall abscess formation.

We report the case of a 68-year-old man who presented with recurrent fever and persistent right thoracic pain for two months, 11 years after laparoscopic cholecystectomy for acute cholecystitis. Computed tomography revealed a large 15 × 11 cm perihepatic collection located between the liver and intercostal muscles, containing a radiopaque foreign body. A limited open approach was performed through a 3-cm incision over the palpable collection. Purulent material was evacuated, and the foreign body was macroscopically identified as a gallstone. The patient remained asymptomatic at one-month follow-up.

Retained gallstones may remain occult for several years and subsequently cause abscess formation in unusual locations. Imaging can provide an important diagnostic clue, particularly when a calcified stone is visible within an abscess. Management depends on the location and depth of the collection and generally requires drainage and removal of the retained stone to achieve definitive source control.

A retained spilled gallstone should be considered in the differential diagnosis of a delayed perihepatic or abdominal wall abscess in patients with a history of laparoscopic cholecystectomy. Careful review of imaging and surgical history may facilitate diagnosis, while complete drainage and retrieval of the retained stone can provide definitive treatment.

## Introduction

Intraoperative gallbladder perforation is a relatively common complication of laparoscopic cholecystectomy, with reported rates generally around 10%-20% [1], although recently published series report rates ranging from approximately 5% to 36% [2]. Gallbladder perforation can result in bile and/or gallstone spillage [1]. Retained spilled stones can rarely cause delayed complications, including perihepatic/subhepatic abscesses. One PubMed study found intra-abdominal abscesses in 2.9% of patients with both bile and gallstone spillage versus 0.6% with bile spillage alone [3]. The clinical presentation of an abscess due to dropped gallstones may be nonspecific and does not readily suggest this etiology. We present a case of a perihepatic abscess occurring 11 years after laparoscopic cholecystectomy.

## Case presentation

A 68-year-old man presented to the Emergency Department with thoracic pain and fever. His medical history was significant for type 2 diabetes mellitus, arterial hypertension, hypercholesterolemia, abdominal obesity, and a previous laparoscopic cholecystectomy for acute cholecystitis. Regarding his current symptoms, the patient had been experiencing persistent thoracic pain for approximately two months. During this period, he had consulted his family physician several times and underwent chest radiography to rule out a rib fracture. In the absence of significant radiological findings, he was treated symptomatically for presumed costochondritis. However, he subsequently developed recurrent episodes of fever, prompting his presentation to the Emergency Department.

A computed tomography (CT) scan of the thoracoabdominal region revealed a large collection measuring 15 × 11 cm, located between the liver and the intercostal muscles. The emergency physician requested a thoracic surgery consultation, and the patient was subsequently referred to the digestive surgery team.

On examination, the patient was afebrile and hemodynamically stable. Abdominal examination revealed a palpable swelling in the right upper quadrant. Laboratory investigations showed mild inflammatory syndrome, with a C-reactive protein level of 39.7 mg/L and leukocytosis of 11.4 × 10⁹/L. However, liver function tests were within normal limits. Table 1 summarizes the laboratory findings.

**Table 1:** Laboratory findings

Biology	Result	Unit	Reference Range
Hemoglobin	14	g/dL	13-18
C-Reactive Protein	39.7	mg/L	<5
White Blood Cells	11.4	× 10⁹/L	4-11
Aspartate Aminotransferase (AST)	30	U/L	10-50
Alanine Aminotransferase (ALT)	39	U/L	10-50
Gamma-Glutamyl Transferase	52	U/L	<60
Alkaline Phosphatase	62	U/L	40-129
Bilirubin	12	µmol/L	<24
Creatinine	98	µmol/L	59-104

Upon reassessment of the CT images, a foreign body was identified within the perihepatic abscess (Figure 1). Further questioning revealed that the patient's previous laparoscopic cholecystectomy in 2015 had been complicated by intraoperative gallbladder perforation and spillage, requiring placement of a surgical drain at the end of the procedure and transfer to the Intensive Care Unit (ICU). His ICU stay was unremarkable, and after stabilization, he was discharged. Based on this history and the CT findings, we hypothesized that a gallstone may have been inadvertently spilled into the peritoneal cavity during the initial procedure and subsequently served as a nidus for abscess formation. 

**Figure 1:**
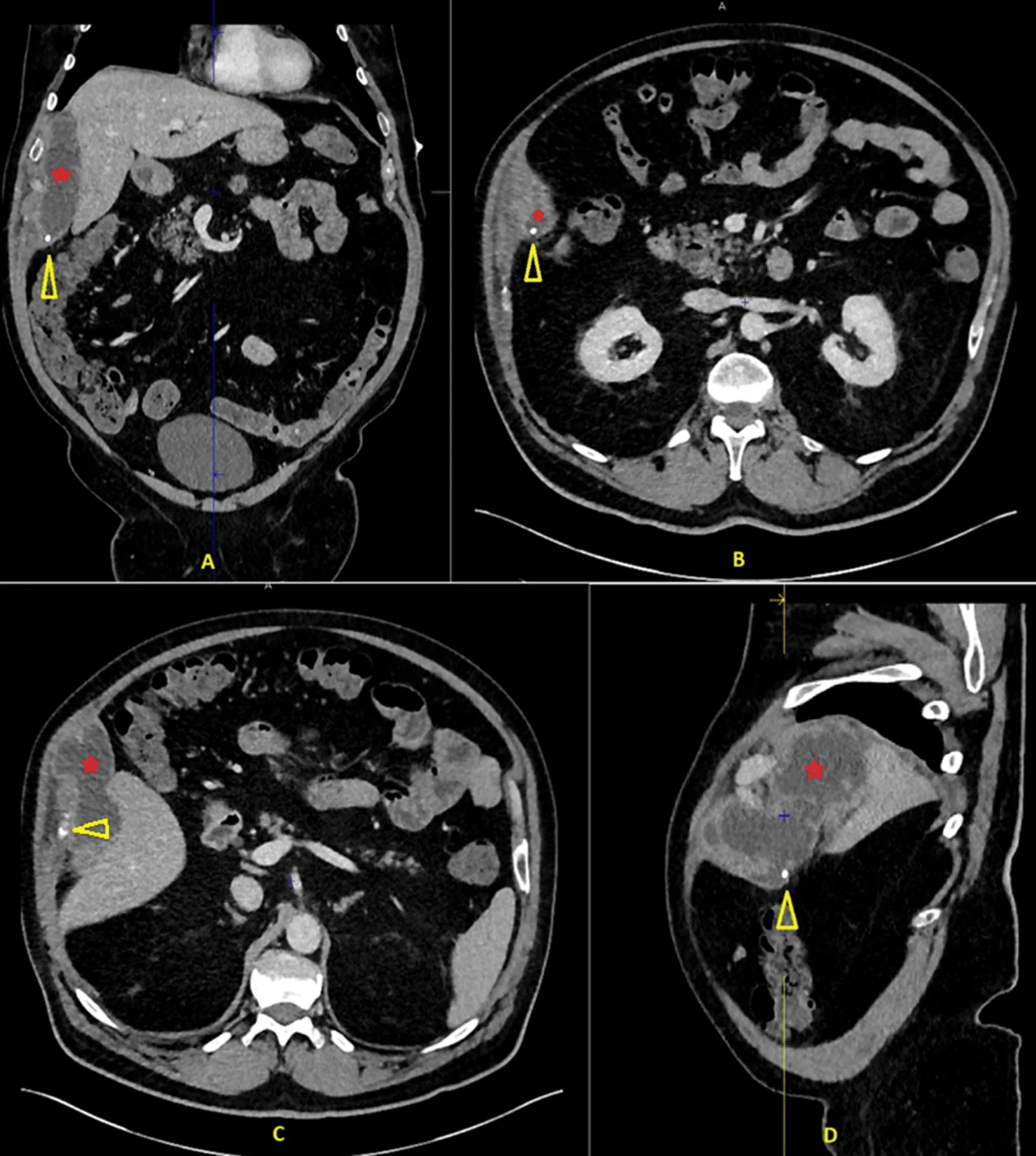
Computed tomography images demonstrating the perihepatic abscess The yellow arrow shows the foreign body corresponding to the gallstone. The red star shows the collection. Panel A: Coronal view showing the calcified foreign body (yellow arrow) inside the collection (red star). Panels B and C: Transverse views showing the calcified foreign body (yellow arrow) and the collection (red star). Panel D: Sagittal view showing the calcified foreign body (yellow arrow) and the collection (red star).

The decision was therefore made to drain the abscess and, if possible, retrieve the foreign body in order to confirm our hypothesis. Despite the patient's abdominal obesity, the collection was palpable on physical examination. We therefore opted for a limited open approach rather than laparoscopy. A small incision was chosen to minimize the invasiveness of the procedure and, importantly, to avoid contamination of the peritoneal cavity with purulent material.

Under general anesthesia, a 3-cm incision was made at the upper border of the right upper quadrant, directly over the palpable collection. Purulent material was evacuated, and the foreign body was digitally identified and retrieved. Macroscopic examination confirmed that the foreign body was a biliary calculus. Specifically, it was a single brown cholesterol stone measuring 4 mm, with a noncalcified macroscopic appearance (Figure 2).

**Figure 2:**
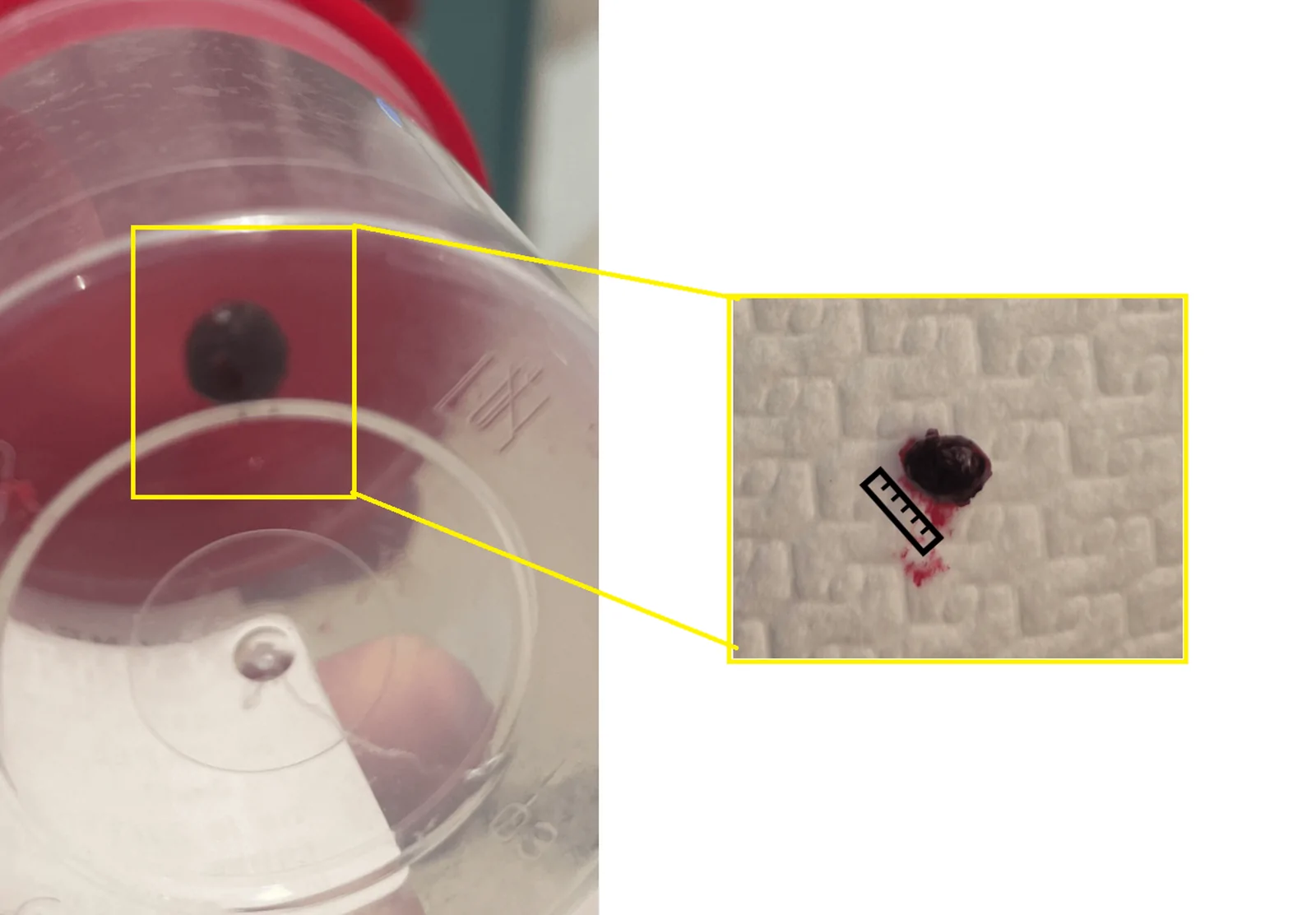
Gallstone retrieved during abscess evacuation, measuring 4 mm

The findings confirmed our hypothesis of a dropped gallstone following the previous laparoscopic cholecystectomy, which had subsequently acted as a nidus for the development of a delayed perihepatic abscess.

The abscess cavity was rinsed with Betadine and sodium chloride, and an aspirative drain was left in the cavity through the same incision. Moreover, the postoperative period was unremarkable; the drain was removed on day 2, and the patient was discharged with symptomatic and antibiotic treatment: paracetamol and amoxicillin/clavulanic acid. Furthermore, the patient was followed up one month after the operation; he was asymptomatic, and the wound healed without complications, as shown in Figure 3. Notably, no imaging was performed in the absence of symptoms.

**Figure 3:**
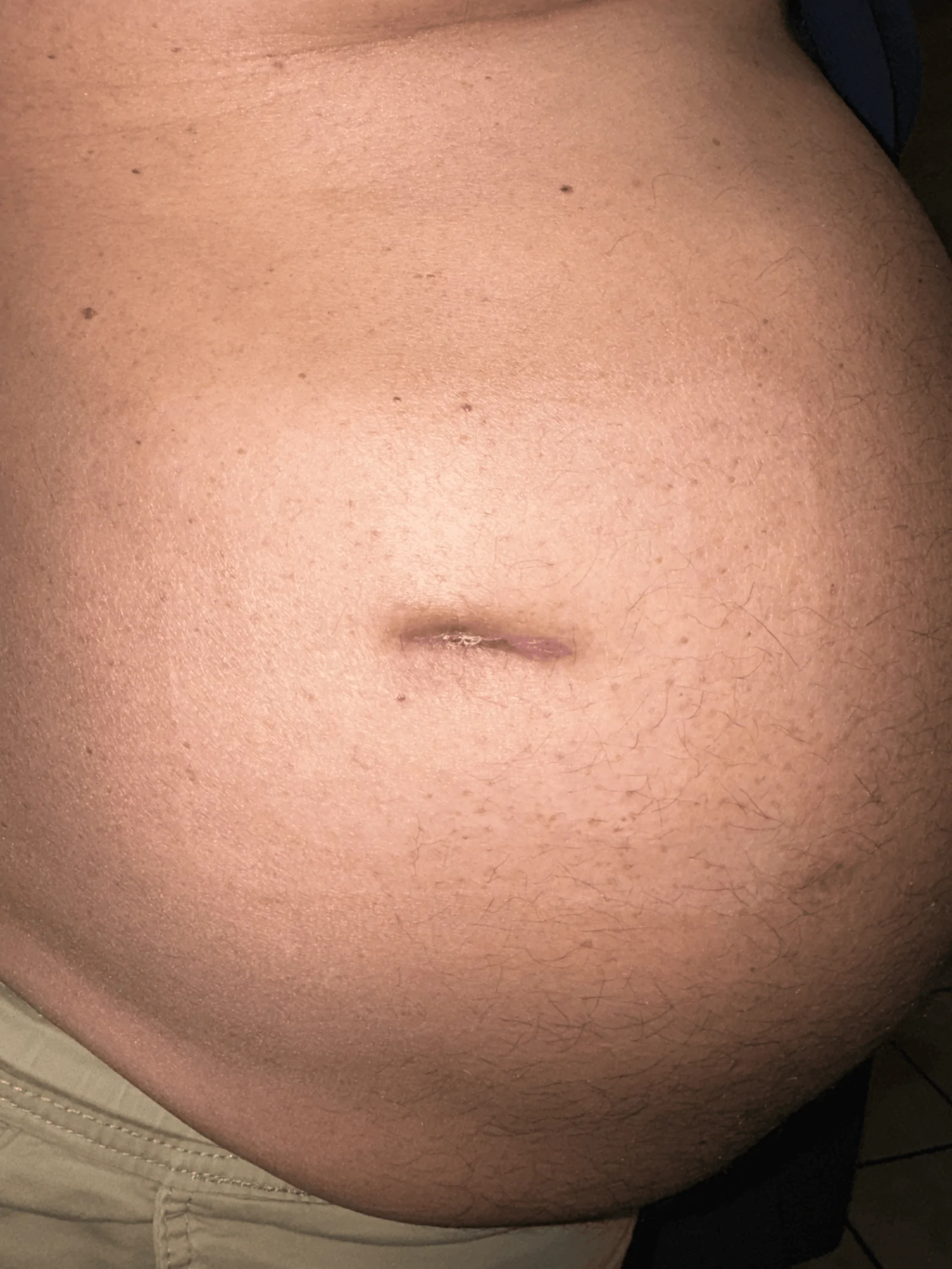
Image showing the incision healing one month after the operation

## Discussion

Dropped gallstones can remain occult for years before presenting as a perihepatic or abdominal wall abscess. Interestingly, Dobradin et al. showed that it may occur even eight years after laparoscopic cholecystectomy [4]. The latency is highly variable. In Table 2, we show the pattern of latency for abscess formation. For instance, some abscesses occur relatively early - within months and particularly during the first year - but there is a well-documented tail extending 5, 8, 10, 15, and even 30 years after laparoscopic cholecystectomy. There is a particularly strong comparison with the 2015 report by Robinson et al. [5], because that case involved a perihepatic abscess five years after laparoscopic cholecystectomy, whereas our case involves a perihepatic abscess 11 years later. Our case therefore falls squarely within the documented phenomenon of very delayed complications.

**Table 2:** Characteristics of reported cases in the literature CT: Computed Tomography; US: Ultrasound; RUQ: Right Upper Quadrant

Reference	Age	Sex	Latency (years)	Abscess location	Diagnostic approach	Treatment	Study design
Frola et al. [6]	74	F	1.5	Intraperitoneal	CT + US	Local incision and drainage	Case report
Hand et al. [7]	50	F	2	Umbilical port-site	CT + US	Local incision and drainage	Case report
Peravali and Harris [8]	61 and 86	M	3 and 5	Perihepatic	CT	Laparoscopic	2 case reports
Awwad et al. [9]	76	F	5	Retroperitoneal	CT	Local incision and drainage	Case report
Rutherford and Malik [10]	74	M	5	Perihepatic	CT	Laparoscopic + Local incision and drainage	Case report
Robinson et al. [5]	53	M	5	Perihepatic	CT	Local incision and drainage	Case report
Singh et al. [11]	42	F	7	Retroperitoneal	CT	Local incision and drainage	Case report
Dobradin et al. [4]	82	M	8	RUQ abdominal wall	CT + US	Local incision and drainage	Case report
Quinn et al. [12]	73	F	10	Intraperitoneal	CT	Local incision and drainage	Case report
Pottakkat et al. [13]	28	F	11	Perihepatic	CT	Local incision and drainage	Case report
Robinson et al. [14]	39	F	12	Anterior abdominal wall	CT	Local incision and drainage	Case report
Oh et al. [15]	73	F	15	Intraperitoneal	CT	Laparoscopic + Local incision and drainage	Case report
Christensen AM and Christensen MM [16]	53	F	17	Pelvic abdominal wall	CT	Local incision and drainage	Case report
Nan et al. [17]	74	F	30	Abdominal wall	CT	Local incision and drainage	Case report
Syed et al. [18]	58	M	8	Perihepatic	CT	Local incision and drainage	Case report
Kumar and Haas [19]	86	M	>15	RUQ abdominal wall	CT	Local incision and drainage	Case report
Present Case	68	M	11	Perihepatic	CT	Local incision and drainage	Case report

Imaging is a clue in the diagnosis; some authors used ultrasound to emphasize that echogenic structures with posterior shadowing within an abscess in a post-cholecystectomy patient can strongly suggest a gallstone-related abscess [4]. In parallel to the five-year perihepatic abscess reported by Robinson et al. [5], we based our diagnosis on CT findings showing a calcified/radiopaque focus within or adjacent to an abscess. However, this is not reliable because many gallstones are radiolucent. Consequently, CT may show only the abscess and not the stone. On the other hand, ultrasound demonstrated echogenic structures with posterior acoustic shadowing within the abscess, which strongly suggested gallstones. In our case, the gallstones were calcified, so we did not find it necessary to postpone the surgery to undergo an ultrasound evaluation. Nevertheless, other reports showed that the diagnosis was only made during surgery [9].

Speaking of the surgical approach to management, the important point is that both laparoscopic and open approaches have been used. Indeed, the cases are heterogeneous, and many were treated by local incision/drainage rather than laparoscopy [4,5,18]. For instance, the 15-year case is probably the most interesting precedent if considering a laparoscopic approach [15]. The patient had an intra-abdominal abscess adjacent to the abdominal wall, and the stone was removed laparoscopically after drainage.

In contrast, the eight-year case reported by Dobradin et al. was much more superficial [4]: the abscess was in the abdominal wall, so they performed a local open incision and drainage, retrieved two stones, irrigated the cavity, and left a drain. There was no need for laparotomy. Moreover, the 2023 eight-year case similarly used an open local operation but went further by excising the chronic abscess cavity/capsule together with the retained spilled stone [18]. Precisely, we opted for open access because the abscess was superficial. Moreover, we considered that controlled evacuation of the abscess using gauze would provide a better opportunity to locate and retrieve the suspected gallstone than simple aspiration of the purulent collection.

This is a single case report that highlights the importance of gallstone-related abscesses and the possibility of long latency. Larger studies are needed to standardize the management of perihepatic abscesses, especially after cholecystectomy.

Written informed consent was obtained from the patient for publication of this case report and the accompanying images. A copy of the written consent is available for review by the Editor-in-Chief of this journal on request. This report was prepared in accordance with the Declaration of Helsinki and local policy on case reports. The consent also covers publication of the images.

## Conclusions

A delayed abscess should prompt consideration of retained spilled gallstones, especially in a patient with a history of cholecystectomy. Ultrasound can provide an important diagnostic clue, as many gallstones are radiolucent. The surgical approach is usually through a local incision; however, a laparoscopic approach may be useful for deeper abscesses.
